# Guided tours in historical and religious sites: emotional restoration and the role of individual differences

**DOI:** 10.3389/fpsyg.2026.1795190

**Published:** 2026-05-28

**Authors:** Laura Miola, Guido Furlan, Greta Montanari, Gianmario Guidarelli, Elena Svalduz, Jacopo Bonetto, Andrea Giordano, Francesca Pazzaglia

**Affiliations:** 1 Department of General Psychology, University of Padova, Padua, Italy; 2 Interuniversity Research Center in Environmental Psychology (CIRPA), Sapienza Università di Roma, Rome, Italy; 3 Department of Cultural Heritage: Archaeology, History of Art, Film and Music, University of Padova, Padua, Italy; 4 Department of Civil, Environmental and Architectural Engineering, University of Padova, Padua, Italy

**Keywords:** historical site, individual differences, nature, religious site, restorativenes

## Abstract

**Introduction:**

This study investigated how guided tours to historical and religious environments, specifically the archaeological site of Aquileia and the Benedictine Abbey of Praglia, both located in north-eastern Italy can influence perceived restorativeness and emotional responses (valence and arousal).

**Methods:**

A total of 149 participants completed questionnaires before and after a 45-min guided tour, assessing demographic variables, religiosity, connectedness to nature, emotional responses, perceived stress over the last month, and restorativeness.

**Results:**

Results revealed that emotional valence increased from pre- to post-visit at both sites, while arousal remained stable at Praglia, but increased at Aquileia. Restorativeness was positively predicted by the significance attributed to the visit, particularly regarding artistic, cultural, and historical values (Praglia and Aquileia), along with individual differences such as connectedness to nature (Aquileia) and perceived stress (Praglia).

**Discussion:**

Overall, visits to religious and historical sites in natural settings enhanced positive emotions, with site-specific differences in arousal. Perceived restorativeness was associated not only with the cultural and historical significance attributed to the visit but also with individual factors such as connectedness to nature and stress levels. These results highlight the role of both contextual meaning and personal characteristics in shaping restorative experiences, offering practical implications for designing guided tours that maximize psychological benefits.

## 1 Introduction

A considerable amount of literature showed that the exposure to nature can show a positive impact on people's wellbeing, restoring psychological resources (Menardo et al., 2021; Gaekwad et al., 2022). The beneficial effects of natural environments are commonly explained within environmental psychology by two major theoretical frameworks, namely the Stress Reduction Theory (SRT; Ulrich, 1983) and the Attention Restoration Theory (ART; Kaplan and Kaplan, 1989). Both perspectives assume that restorative processes are activated when individuals are in a state of psychophysiological stress or attentional fatigue, that is, when available resources are insufficient to cope with environmental demands (Kaplan and Kaplan, 1989; Ulrich et al., 1991; Ulrich, 2023).

SRT emphasizes the role of rapid, automatic affective responses to environmental stimuli. According to this framework, exposure to natural settings elicits an immediate, pre-conscious affective appraisal—typically characterized by positive valence—that reflects an evolved sensitivity to supportive and non-threatening environments. This initial affective response is accompanied by coordinated physiological changes, including a reduction in autonomic arousal and a shift toward parasympathetic activation. Over time, these early affective–physiological processes become accessible at a conscious level, resulting in a subjectively experienced improvement in mood and a decrease in perceived stress. Thus, within SRT, restoration unfolds from fast, automatic affective reactions toward more stable and consciously reportable emotional states.

In contrast, ART focuses on the recovery of cognitive resources, particularly directed attention, which becomes depleted after prolonged, effortful engagement with demanding tasks. Natural environments are considered restorative because they preferentially engage involuntary attention through “soft fascination,” allowing the executive, effortful attentional system to rest and replenish. As attentional capacity is restored, individuals regain the ability to effectively regulate thoughts and actions, which may also indirectly contribute to improvements in affective experience and perceived stress.

According to ART, restorative environments are characterized by four key properties. Being Away refers to the experience of psychological or physical distance from routine demands and stressors. Fascination denotes the presence of stimuli that effortlessly capture attention without requiring cognitive control. Extent describes the sense of immersion in a coherent and sufficiently rich environment that sustains engagement. Finally, Compatibility refers to the fit between environmental affordances and the individual's purposes, inclinations, and needs, allowing for smooth and effective interaction with the setting. Together, these properties define the environmental conditions under which attentional recovery—and, more broadly, restorative experience—can occur.

More recent theoretical models on the relationship between nature and wellbeing have highlighted the importance of *nature experience*, characterized by the specific ways in which individuals actually interact with nature and the different levels of awareness and perception of natural environments—often referred to as the *internal dose*. The latter may vary according to factors such as attention, personal preferences, a sense of connection with nature, and individual attitudes (Bratman et al., 2019). Therefore, these models suggest that the environment, the experiences within it, and individual factors all jointly contribute to restorative processes.

The literature on restorativeness has mainly focused on natural environments, while less is known about the relationship between individuals and built or mixed environments (those combining natural and constructed features). Studies have often used built environments as a control condition when investigating the benefits of nature, frequently selecting spaces that are deliberately unattractive, monotonous, or gray (see Weber and Trojan, 2018; for a review). By contrast, recent research on built environments with historical, artistic, or religious value—such as monasteries, museums, or historic districts—has begun to highlight their restorative potential (Bornioli et al., 2018).

Among historical environments, several studies have focused on religious settings. These have been examined mainly within the psychology of religion, exploring the effects of spirituality or specific retreats on individuals' wellbeing and health. For example, Ouellette et al. (2005) asked participants attending religious retreats at a Canadian abbey to report their health issues (such as work-related or alcohol problems), motivations for attending, and activities during their stay. They found that health problems predicted both the motivation to attend and engagement in activities such as praying and resting. In another study on spiritual retreats, participants evaluated their perceived restorativeness, overall experience (such as feelings of peacefulness, connectedness, and spiritual engagement), and relaxation and recovery states. The results showed that being mentally away, participating in spiritual activities, and refraining from technology predicted both immediate and enduring restorative outcomes (Gill et al., 2019). In a study conducted in Romania, researchers analyzed 416 TripAdvisor reviews of monasteries. The content analysis identified ten recurring themes (such as *monastery, painted, tower, visit, beautiful, inside, famous, place, blue*, and *guide*) describing key elements of visitors' experiences. The results highlighted the importance of both religious and non-religious aspects—such as architecture, aesthetics, history, people, and the general visiting experience (Lupu et al., 2019).

Although some studies have investigated built religious environments, relatively few have explored how individual spirituality might influence the restorative qualities of visitors' experiences. For instance, a study conducted during the COVID-19 pandemic found that perceived environmental restorativeness associated with a pilgrimage site positively influenced spirituality, which in turn improved pilgrims' psychological wellbeing (Kim et al., 2024). Another study examined these aspects independently of the specific characteristics of a place, showing that spirituality mediates the relationship between connectedness to nature and psychological wellbeing (Kamitsis and Francis, 2013).

Regarding historical places more generally (not limited to religious contexts), a study on cultural heritage sites in South Korea found that perceived restorativeness and emotional healing were key factors influencing both visitor satisfaction and the intention to revisit (Cho et al., 2016). Similarly, a study on a historical district in China showed that visitors' landscape perception significantly influenced *place dependence*, suggesting that individuals form emotional connections and attachments based on perceptions of aesthetic, historical significance, uniqueness, pleasantness, natural elements, and safety. Moreover, that perception could directly trigger restorative experiences and indirectly contribute to emotional identification (Li et al., 2023).

Other studies have investigated the restorative value of museums as artistic environments and their effects on visitors. For example, one study found that physiological responses during art viewing were significantly linked to aesthetic–emotional experiences. Specifically, the dimensions of *Aesthetic Quality, Surprise/Humor, Dominance*, and *Curatorial Quality* were associated with heart rate (HR), HR variability, and skin conductance variability (Tschacher et al., 2012). Similarly, Mastandrea et al. (2019) compared figurative and modern art exhibitions with a control condition in terms of blood pressure (BP) and HR after the visit. Participants in the figurative-art condition exhibited BP reductions, whereas only one-third of those in the modern-art condition showed decreases. In a second study comparing ancient and modern art museums, the authors found that visitors to the ancient art museum primarily sought understanding and knowledge, while visitors to the modern art museum engaged more from an emotional and pleasure-oriented stance (Mastandrea et al., 2009).

As evidenced by this review, research investigating how individuals interact with religious, artistic, and historical buildings is heterogeneous. Studies differ both in the variables examined and, in the measures employed. Some have focused on visitors' motivations and onsite activities, while others focus on perceived restorativeness, relaxation, and emotional effects; still others on environmental perception, landscape attributes, and place identity. A few studies have also incorporated spirituality within religious contexts.

In summary, the literature on religious and historical environments emphasizes that experiences, emotions, and the meanings people attribute to these places play a crucial role in their appreciation. However, it remains unclear whether, and to what extent, these factors are relevant for restorativeness in such settings. Therefore, the present study aims to investigate exposure to historical and religiously connoted environments, jointly considering individual differences, visitors' perceived significance of the experience, emotional valence and arousal, and perceived restorativeness.

For this purpose, this study selected two sites of high historical and cultural value: the Abbey of Praglia (a historical and religious environment) and the archaeological area of Aquileia (a historical environment), both situated within natural contexts that contribute to creating a compelling and evocative atmosphere.

To operationalize the core constructs derived from SRT (Ulrich, 1983; Ulrich et al., 1991) and ART (Kaplan and Kaplan, 1989) within a naturalistic field setting, the present study relies on subjective indicators of affective and restorative experience. In line with SRT, which posits that exposure to restorative environments elicits rapid affective responses accompanied by changes in physiological arousal, participants' affective states were assessed before and after the visit using the Self-Assessment Manikin (SAM). This measure captures perceived valence and arousal, thus providing an indirect but theoretically grounded index of the affective–physiological shifts associated with stress reduction. Although SRT emphasizes early, automatic affective processes, self-reported affect represents their conscious and stabilized outcome, and has been widely employed in empirical studies adopting this framework (e.g., Ulrich et al., 1991).

With respect to ART, which emphasizes the recovery of directed attention through exposure to environments rich in softly fascinating stimuli, direct cognitive performance measures were not feasible in the present field-based design. Therefore, perceived restorativeness was assessed using the Perceived Restorativeness Scale (PRS-11, Pasini et al., 2014), a validated instrument capturing the extent to which an environment is experienced as restorative in terms of its key components (e.g., being away, fascination, coherence, compatibility). While the PRS does not measure attentional recovery *per se*, it provides a well-established proxy of the environmental qualities that support such recovery and has been extensively used in applied and ecological research.

In addition, individual differences were assessed to account for variability in restorative responses. These included measures of nature connectedness and religious attitudes, which may modulate sensitivity to environmental affordances, along with perceived stress in the previous month, conceptualized as an indicator of baseline resource depletion. Finally, participants' subjective evaluations of the value attributed (significance) to the two visited environments (such as natural, historical, and artistic qualities) were collected to capture the multidimensional nature of real-world restorative settings. Together, these measures allow for an integrative examination of affective and restorative processes in complex, ecologically valid contexts, without assuming a strictly unidirectional or mechanistic causal sequence between them.

Based on SRT (Ulrich, 1983; Ulrich et al., 1991), it is expected that exposure to the visited environments may elicit improvements in participants' affective states. Specifically, H1 predicts that emotional valence may increase and perceived arousal may decrease from pre- to post-visit across both sites, reflecting a shift toward a more positive and less activated affective state.

With regard to individual differences, H2 posits that higher levels of perceived stress in the previous month may be associated with stronger restorative effects. In particular, individuals reporting higher stress are expected to show greater increases in positive valence, greater reductions in arousal, and higher perceived restorativeness (PRS), consistent with the assumption that individuals experiencing greater resource depletion benefit more from restorative environments.

Similarly, H3 predicts that greater connectedness to nature may be associated with enhanced restorative outcomes across both sites (given mixed natural and built components), both in terms of affective change (SAM) and perceived restorativeness (PRS), reflecting increased sensitivity to environmental affordances.

Differences between the two sites are addressed in H4. Given the distinctive characteristics of Praglia Abbey, which include stronger spiritual and religious connotations, the hypothesis is expected that participants who attribute greater value to spiritual dimensions will report higher perceived restorativeness in Praglia.

Finally, H5 concerns the role of subjective environmental appraisal. This hypothesis is expected that participants' valuation of specific environmental qualities, namely natural, historical, artistic, and religious aspects, may differentially predict restorative outcomes across the two sites. In particular, natural qualities are expected to contribute to restorative responses in both contexts, whereas historical and artistic evaluations are anticipated to play a stronger role in Aquileia, and religious/spiritual evaluations in Praglia. These dimensions are thus hypothesized to act as context-sensitive modulators of both affective change and perceived restorativeness.

## 2 Materials and method

### 2.1 Demographic questionnaire

Questionnaire with questions on gender, age, education level, and occupation.

### 2.2 Self-Assessment Manikin

SAM (Bradley and Lang, 1994) is a non-verbal tool used to measure emotional responses. It consists of stylized figures of a human figure that graphically represent different emotional intensities, allowing participants to self-assess their emotional state without verbal descriptions. The questionnaire assesses two emotional dimensions: pleasure/displeasure (valence) and activation/deactivation (arousal). Participants are asked to answer the question, “How do you feel right now?” using a 9-point scale, where 1 represents the lowest value for each dimension (negative valence or low arousal) and 9 the highest (positive valence or high arousal).

### 2.3 Santa Clara Strength of Religious Faith

The Santa Clara Strength of Religious Faith (SCSRF; Plante and Boccaccini, 1997) is a 10-item questionnaire designed to evaluate the depth and strength of the participant's perceived relationship with God or their religious faith. Each item is rated on a Likert scale from 1 (strongly disagree) to 4 (strongly agree), with statements related to spirituality and emotional support derived from faith. An example item is: “My relationship with God helps me not to feel lonely.” The SCSRF scale showed excellent internal consistency (Cronbach's α = 0.977).

### 2.4 Connectedness to nature scale

CNS (Mayer and Frantz, 2004) includes 14 items aimed at assessing the extent to which participants feel connected to the natural environment. The questions investigate how much participants feel part of nature and their appreciation of this bond. Each item is rated on a Likert scale from 1 (not at all true) to 5 (completely true). An example item is: “I often feel as though I am a small part of the natural world around me, no more important than the grass on the ground or the birds in the trees.” The CNS demonstrated good internal consistency (Cronbach's α = 0.809).

### 2.5 Perceived stress scale

The perceived stress scale (PSS; Cohen et al., 1994) is an instrument used to measure the degree to which life situations in individual's life during the *last month* are subjectively perceived as stressful. It focuses on how unpredictable, uncontrollable, or overwhelming participants find their lives in the previous month. The questionnaire consists of 10 items assessing stress perception over the past month. Each item is rated on a Likert scale from 0 (never) to 4 (very often). An example item is: “In the last month, how often have you felt nervous or stressed?” The PSS showed good internal consistency (Cronbach's α = 0.824).

### 2.6 Perceived Restorativeness Scale

The Perceived Restorativeness Scale-11 (PRS-11; Pasini et al., 2014) evaluates the extent to which an environment promotes psychological restoration and recovery. This shortened version of the PRS is based on ART. Perceived environmental restorativeness is measured across four factors with 11 items: three for Being Away, three for Fascination, three for Coherence, and two for Scope. The questionnaire provides an overall Restorativeness score and scores for each subscale. Items are rated on a Likert scale from 0 (not at all) to 10 (very much). An example item is: “In places like this, my attention is drawn to many interesting things.” PRS demonstrated good internal consistency (Cronbach's α = 0.850).

### 2.7 *Ad hoc* questions on the visit experience

In order to explore participants' subjective experience of the visit, this study developed a set of *ad hoc* questions. Respondents were asked to indicate, on a 5-point Likert scale (0 = not at all and 4 = very much), the extent to which specific features of the site contributed to making the visit meaningful. The items covered four main aspects, namely (1) being in a place rich in art and culture [Art and Culture]; (2) being in a place with religious and spiritual references [Religiosity]; (3) being in an ancient place rich in history [History and Antiquity]; and (4) being in a place immersed in a wide and lush natural setting [Nature].

Participants were also asked to rate, again on a 5-point Likert scale, the extent to which they appreciated specific opportunities offered by the visit, including (1) admiring the surrounding landscape; (2) enjoying a relaxing break; (3) deepening their knowledge of the site's historical importance; (4) appreciating the architectural features of the site; and (5) experiencing contact with monastic life.

### 2.8 Site descriptions

The research was conducted at two distinct locations where the above questionnaires were administered.

#### 2.8.1 Praglia Abbey

**Figure 1:**
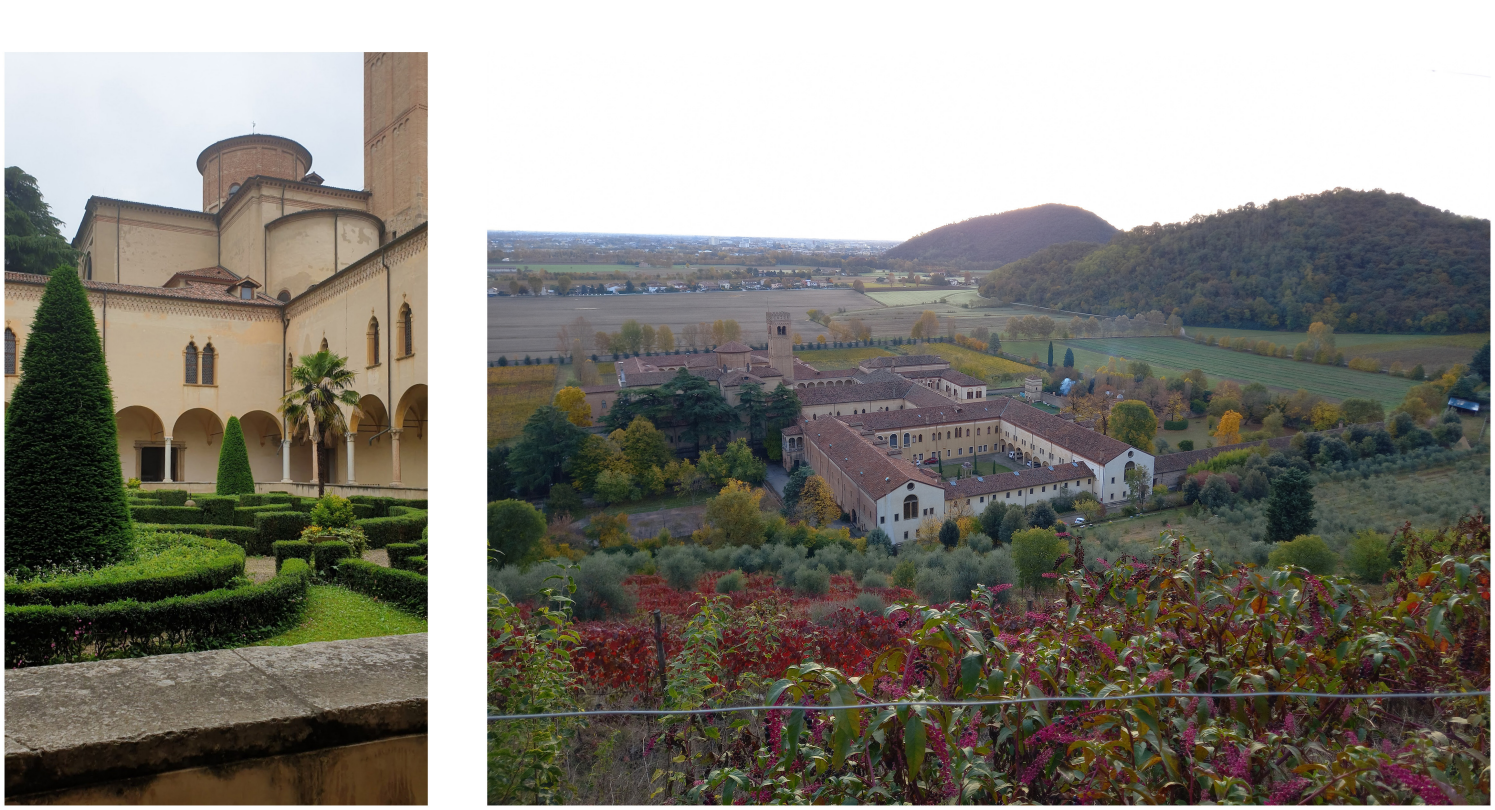
Images of Praglia Abbey. Aerial view of the Abbey and photograph of the Abbey Cloister.

Praglia Abbey (see Figure 1) was founded between the late 11th and early 12th centuries on the northern slopes of the Euganean Hills, along the ancient Montanara road from Padua to Este (Ceschi et al., 2013). The monastery was built by reusing a pre-existing castle donated by the Maltraversi family of Montebello and was initially dependent on the monastery of San Benedetto di Polirone near Mantua. By the 14th century, the Praglia community became autonomous, electing its own abbot.

After joining the reform of St. Justine in 1448, the Abbey was rebuilt, replacing the medieval structures, of which only the bell tower remains, and completed with the construction of the new church in 1548. The following centuries marked a period of prosperity until the Napoleonic suppression in 1810. Although the monks returned in 1834, the community was again dissolved in 1867 due to a new suppression law. Most monks relocated to Daila in Istria, while a few remained as custodians. Monastic life finally resumed permanently in 1904 and continues to this day.

#### 2.8.2 Archaeological site of Aquileia

**Figure 2:**
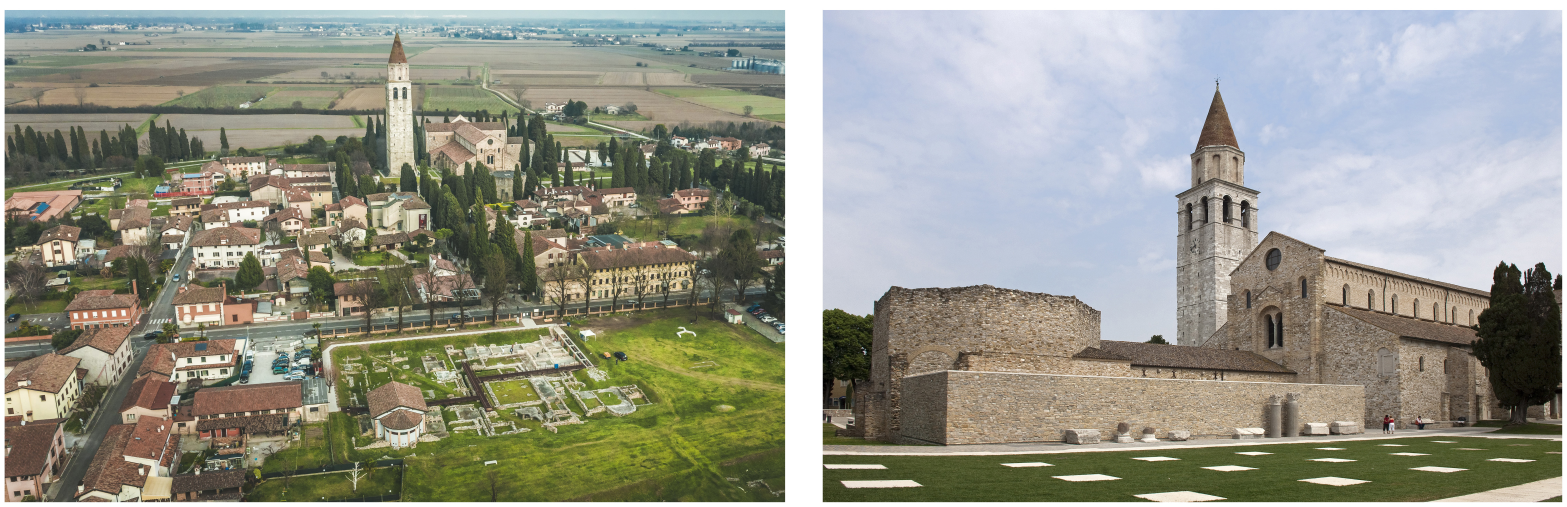
Images of archaeological site of Aquileia. The archaeological area of the CAL plots within the current urban fabric of Aquileia and the southern hall of the baptistery and the basilica of Aquileia seen from the south. Photo by G. Baronchelli from Tiussi (2024).

Aquileia (UD, see Figure 2) is a small town in the Friulian plain, founded as a Latin colony in 181 BC. During the Roman era, it became a major imperial metropolis (Ghedini et al., 2009), thanks to its strategic location and efficient river port system, serving as a key trade hub for goods from the Eastern Mediterranean. This prosperity led to extensive urban and monumental development, including public buildings such as forums, theaters, amphitheaters, baths, and richly decorated private homes with elaborate mosaics.

By the early 4th century AD, Aquileia emerged as one of the first Christian centers, marked by the construction of a grand episcopal and basilica complex. However, from the late 5th century onward, the city declined and transformed into a small medieval village centered around its surviving basilica.

In present days, the ancient city area combines archaeological remains, green spaces, and modern districts with both residential and commercial functions.

### 2.9 Procedure

The research was conducted across multiple days in two distinct sites: the archaeological area of Aquileia and the Abbey of Praglia. Participants completed the following questionnaires, namely:the Demographic Questionnaire; SAM; SCSRF; CNS; PSS in the last month. Subsequently, the participants joined a 45-min tour of the site (Praglia or Aquileia). After the guided tour, participants completed again the SAM and the Perceived Restorativeness Scale (PRS-11) and additional questions about their experience about the visit. The entire participation, including questionnaire completion and guided tour, lasted approximately 1 h per participant.

### 2.10 Participants

The study included a total of 149 participants (77 from Aquileia and 72 from Praglia), who were individuals spontaneously visiting the two sites and were invited on-site to take part in the study while they were waiting for their visit. The average age of the participants was 51.2 years (SD = 14.09), with an age range from 19 to 82 years. All participants provided informed consent before participating in the study. The research protocol was approved by the local ethical committee of the University of Padova (protocol number: 2024_251R1). An *a priori* power analysis was conducted using the pwr package in R to determine the minimum sample size required for the multiple linear regression analysis. We estimated the sample size necessary to detect a medium effect size (Cohen's *f*^2^ = 0.15) for the addition of a single predictor to a model containing up to eight variables with a significance level (α) of 0.05 and a statistical power of 0.80. The analysis indicated that a minimum of 62 participants was required to achieve adequate power.

### 2.11 Statistical analysis

All the analyses were run with Rstudio. First, this study computed descriptive statistics for key variables separately for the Aquileia and Praglia samples and compared using the independent samples *t*-tests. Effect sizes were estimated with Cohen's *d*, and confidence intervals (CI) were reported to assess the magnitude of differences. Subsequently, this study conducted the Pearson's correlations to examine associations among demographic variables, individual factors, restorativeness, and perceived significance dimensions separately for the Aquileia and Praglia samples (see Supplementary material—Part 2).

Linear mixed-effects models examined the relations between SAM valence and arousal scores in both samples, with individual differences including age, PSS, CNS, religiosity (SCSRF), and the dimensions of the visit's perceived significance (Art and Culture, Heligiosity, History and Antiquity, and Nature). In the analysis, the variable Time was also included, considering the values obtained from the SAM before and after the visit. Participants were included as random factor. Estimates (β), standard errors (SE), degrees of freedom (df), *t*-values, *p*-values, and 95% confidence intervals were provided.

Multiple regression analyses were conducted separately for each sample to examine the relationships between PRS and its four dimensions (being away, fascination, coherence, and scope) with individual differences, including age, PSS, CNS, religiosity (SCSRF), and different facets of perceived significance (art and culture, religiosity, history and antiquity, and nature). Model selection was guided by the Akaike Information Criterion (AIC). Starting from a baseline model including a single predictor or the intercept only, additional predictors were included iteratively. A predictor was retained in the model if its inclusion decreased the AIC by at least two units, following a commonly used threshold for meaningful improvement in model fit (see Tables S1–S10 in Supplementary material). This procedure allowed for identification of models that were both parsimonious and explanatory. Model fit was evaluated using adjusted *R*^2^, AIC, and BIC values.

## 3 Results

### 3.1 Comparisons between Praglia and Aquileia sample

No significant differences were observed in individual characteristics measured before the visit. Specifically, no differences emerged in perceived stress, age, education, and levels of valence and arousal before the tours between the Praglia and Aquileia participants (see Table 1). Only one difference emerged between groups, as Praglia participants exhibited significantly higher religiosity (SCSRF; *t* = −3.09, *p* = 0.002, Cohen's *d* = −0.51, 95% CI = −0.83, −0.18).

**Table 1:** Descriptive statistics of variables measured by the study and *t*-test results.

Variables	Aquileia		Praglia		*t-*Value	*p-*Value	Cohen's *d*	95% CI
	Means	SD	Means	SD				
Age	50.9	15.0	51.5	13.1	−0.26	0.78	−0.04	−0.37, 0.28
Education	3.78	0.805	3.58	0.884	1.41	0.160	0.23	−0.09, 0.55
Connectedness to nature (CNS)	37.6	10.0	40.6	7.84	−1.98	0.05	−0.32	−0.65, 0.00
Religiosity (SCSRF)	18.2	10.1	23.3	10.3	−3.09	0.002	−0.51	−0.83, −0.18
Perceived stress (PSS)	18.4	6.31	19.7	6.63	−1.16	0.24	−0.19	−0.51, 0.13
Valence (PRE)	7.38	1.24	7.08	1.11	1.52	0.12	0.25	−0.07, 0.57
Valence (POST)	7.85	1.31	7.40	1.10	2.21	0.02	0.37	0.04, 0.70
Arousal (PRE)	6.00	1.72	5.58	1.52	1.57	0.11	0.26	−0.07, 0.58
Arousal (POST)	6.66	1.94	5.82	1.84	2.66	0.008	0.45	0.11, 0.78

### 3.2 Linear mixed effect models on emotional valence and arousal (SAM)

For the Praglia sample, the best-fitting model for valence included only the significance attributed to the experience. Specifically, Art and Culture significance emerged as a positive predictor of Valence (β = 0.30, *p* = 0.009), suggesting that participants who attributed greater cultural and artistic meaning to the visit reported more positive emotional evaluations.

Regarding Arousal, the final model included both Art and Culture and Religiosity. The results showed that Religiosity was a significant positive predictor of Arousal (β = 0.29, *p* = 0.008), indicating that participants who ascribed greater religious significance to the experience reported higher levels of activation or emotional intensity.

In the Aquileia sample, the best-fitting model for Valence retained both the significance of the visit and the time factor. Nature was a positive predictor of Valence (β = 0.25, *p* = 0.0007), suggesting that perceiving the visit as meaningful in relation to nature was associated with more positive affective evaluations. Furthermore, the analysis revealed a significant effect of Time (β = −0.32, *p* = 0.012), indicating an increase in Valence from pre- to post-visit—that is, participants reported higher levels of positive affect after the visit.

Regarding Arousal, a significant main effect of Time was observed (β = −0.34, *p* = 0.002), indicating that arousal levels increased from pre- to post-test, suggesting less relaxed emotional state after the visit.

Full tables for the mixed models can be found in the Supplementary material (Part 1).

### 3.3 Multiple regression analysis on perceived restorativeness (PRS) and its dimensions

To examine the contextual and individual predictors of perceived restorativeness, separate multiple regression analyses were conducted for the two sites (Praglia and Aquileia), in line with H4 and H5.

In the Praglia sample, the PRS Total was significantly predicted by participants' appraisal of Art and Culture (β = 0.49, *p* < 0.001), followed by Religiosity (β = 0.25, *p* < 0.05), with the final model explaining a substantial proportion of variance (*R*^2^adj = 0.32). This pattern is consistent with the hypothesis that site-specific qualities—particularly those related to the spiritual and cultural dimensions of Praglia—play a central role in shaping restorative experience.

When considering the PRS subdimensions, differentiated patterns emerged. Being Away was predicted by both Art and Culture (β = 0.45, *p* < 0.001) and Religiosity (β = 0.25, *p* < 0.05), mirroring the pattern observed for the overall score. Scope was exclusively predicted by Art and Culture (β = 0.47, *p* < 0.001), whereas Fascination was primarily associated with History and Antiquity (β = 0.41, *p* < 0.05). Finally, Coherence was uniquely predicted by Nature (β = 0.29, *p* < 0.05). Overall, these findings suggest that in Praglia, different environmental meanings selectively contribute to distinct restorative components, with cultural and religious appraisals playing a particularly prominent role.

In the Aquileia sample, the overall restorative experience (PRS Total) was predicted by both Art and Culture (β = 0.32, *p* = 0.003) and connectedness to nature (CNS_TOT; β = 0.23, *p* < 0.05), with the model explaining a moderate portion of variance (*R*^2^adj = 0.23). In line with H3, individual differences in connectedness to nature contributed to perceived restorativeness in this context, alongside environmental appraisal.

At the level of PRS subdimensions, Being Away was positively predicted by Art and Culture (β = 0.26, *p* < 0.05), CNS_TOT (β = 0.25, *p* < 0.05), and PSS_TOT (β = 0.21, *p* < 0.05), partially supporting H2. Coherence was predicted by both Art and Culture (β = 0.31, *p* = 0.005) and Nature (β = 0.26, *p* < 0.05), indicating a joint contribution of cultural and natural appraisals. Fascination was solely predicted by Art and Culture (β = 0.37, *p* = 0.001), whereas Scope was uniquely associated with connectedness to nature (β = 0.30, *p* < 0.01).

Taken together, these results support the hypothesis that restorative experience is shaped by a combination of environmental appraisal and individual differences, with context-specific patterns emerging across sites. In particular, cultural and artistic evaluations consistently predicted restorative outcomes in both environments, while religious significance played a distinctive role in Praglia, and nature connectedness showed a stronger contribution in Aquileia.

## 4 Discussion

The present study investigated how guided tours in two distinct environments—Aquileia (a historical site) and Praglia (a historical and religious site)—were related to perceived restorativeness and emotional responses (Valence and Arousal). Additionally, this study examined whether individual differences such as age, perceived stress, religiosity, connectedness to nature, and the personal significance attributed to the experience influenced perceived restorativeness and emotional reactions following the tours.

The results showed no significant differences between the Aquileia and Praglia groups in terms of age, education, perceived stress over the previous month, or emotional states before the visits. These findings confirm that there were no baseline differences between groups that could confound the comparison. The only notable difference concerned religiosity, with the Praglia group exhibiting significantly higher levels. This is unsurprising, given that Praglia is a religious site that naturally attracts individuals with stronger religious beliefs and faiths.

Regarding the emotional features of the visits, the analyses revealed divergent patterns between the two sites. In the Aquileia sample, valence and arousal both increased from pre- to post-visit, partially in accordance with H1: the factor Time was statistically significant for both valence and arousal, indicating a general improvement in mood and greater emotional activation. Positive affect was also predicted by Nature, suggesting that the combination of archaeological contents and the surrounding natural environment enhanced positive emotions. Furthermore, arousal levels increased after the visit, implying that the archaeological context—especially when perceived as connected to nature—acted as a stimulating and positively activating setting rather than one inducing relaxation (H1).

Conversely, the emotional response in the monastic complex (Praglia) appeared to depend more on the meanings attributed to the experience rather than on the experience itself. In the Praglia sample, in fact, Time was not a significant predictor for either emotional dimension, suggesting that the visit did not automatically result in an emotional shift for all participants (in contrast with H1). One possible explanation is that the Praglia experience was intimate, involving interior areas of the monastery, within a context characterized by silence and spirituality, which may not elicit the same emotional response in all visitors. In this context, the emotional response was, in fact, strongly conditioned by the meanings attributed to the experience. Valence was predicted by the significance of Art and Culture, indicating that mood improvement occurred primarily for those who deeply appreciated the abbey's artistic and cultural value. Arousal, in contrast, was mainly driven by Religiosity. This challenges the notion of monasteries as purely “passive” or tranquil settings: for visitors attributing high spiritual significance, the experience may elicit active engagement or, as discussed below, feelings of awe (Keltner and Haidt, 2003).

Linear models in the Aquileia sample, indicated that Art and Culture predicted the overall restorative score and Fascination. The sense of Being Away was predicted by a combination of Art and Culture, PSS, and Connectedness to Nature (CNS), indicating that disengagement in an archaeological context is a layered experience influenced by both situational and individual factors (in accordance with H2 and H3). Notably, the perception of Scope—the feeling of being part of something larger—was predicted exclusively by Connectedness to Nature, consistent with previous research (Barbiero and Berto, 2018) showing that individuals with a stronger bond to nature experience a greater sense of environmental unity. Lastly, Coherence was jointly supported by Art and Culture and Nature, confirming the interplay between cultural and natural appraisals.

In the Praglia sample perceived restorativeness was primarily predicted by the subjective meanings assigned to the visit. Specifically, the significance attributed to Art and Culture emerged as the predictor of the overall restorative outcome (PRS Total) and the sense of Being Away, accompanied by Religiosity (in accordance with H4). Thus, in the monastic context, restoration seems deeply intertwined with appreciation of artistic beauty and spiritual connection. A more nuanced picture emerged for the subscales: Scope was driven by Art and Culture, Fascination was uniquely predicted by History and Antiquity, suggesting that historical depth captures involuntary attention, and Coherence was best explained by Nature, indicating that natural elements help structure the environment perceptually and emotionally.

In summary, perceived restorativeness emerged as being shaped by distinct mechanisms across the two sites. In Praglia, restorative outcomes and emotional responses were mainly driven by the meanings attributed to the visit—particularly Art and Culture *and* Religiosity—highlighting a meaning-dependent process. In Aquileia, Art and Culture also played a key role, but restoration appeared more multifaceted, involving Perceived Stress and Connectedness to N*ature*. Moreover, Aquileia showed general increases in mood and arousal over time, whereas in Praglia, emotional changes depended more on individual interpretations and spiritual engagement.

This study acknowledged several limitations. First, the absence of a control group limits the ability to attribute observed pre–post changes in emotional responses directly to the guided tours rather than to time-related or expectancy effects. Future studies should include a control condition—such as participants spending comparable time in a neutral or alternative setting—or ideally, an active comparison group (such as visitors exploring the same sites independently). Second, the specific characteristics of the sample and sites might restrict generalizability. Third, the cross-sectional design limits causal inference. Addressing these issues would strengthen future research. Longitudinal designs may test the durability of restorative effects in religious and historical environments. Studying a broader range of cultural contexts may enhance ecological validity. Furthermore, exploring spirituality, meaning-making, and personal significance as mediators may clarify the mechanisms driving restorativeness.

Another limitation concerns the absence of measures assessing aesthetic sensitivity or aesthetic experience. Since appreciation of artistic and architectural beauty emerged as a main predictor of restoration, including such variables may provide greater insight into individual differences. Similarly, the lack of data on participants' prior familiarity or expertise in art, history, or archaeology may have overlooked influences on engagement and interpretation.

Further, the feeling of awe may be introduced in future studies as a key to interpreting the increase in arousal from pre- to post-visit. Considering the ambivalent concept of awe—which combines positive feelings (wonder, admiration, and fascination) with elements of fear or overwhelm, especially when the experience evokes magnitude or transcendence—it may be hypothesized that such experiences could increase arousal rather than exclusively evoke calm or relaxation (Chirico and Gaggioli, 2018).

Finally, the frequency of visits to comparable settings (such as museums, archaeological sites, or religious monuments) was not assessed; habitual visitors may respond differently from first-time guests, either showing decreased sensitivity due to habituation or deeper engagement through accumulated knowledge.

## 5 Conclusions

Overall, the findings contribute to the growing evidence that cultural and historical settings can promote psychological restoration and positive affect. These results underscore the importance of integrating subjective experience and contextual variables into the environmental restorative study. The findings also suggest practical implications for designing guided experiences that foster wellbeing, offering insight into how historical and religious sites may be curated to maximize their restorative potential.

## Data Availability

The datasets presented in this study can be found in online repositories. The names of the repository/repositories and accession number(s) can be found below: https://zenodo.org/communities/inest_spoke4; https://zenodo.org/records/19582749.
