# Supplementary material for: Guided tours in historical and religious sites: emotional restoration and the role of individual differences
Source: Front Psychol. 2026 May 28;17:1795190. doi: 10.3389/fpsyg.2026.1795190 (PMC13253273; doi:10.3389/fpsyg.2026.1795190)
Supplement: Supplementary file 1 [file Table_1.docx]

Supplementary Material

**Guided Tours in Historical and Religious Sites: Emotional Restoration and the Role of Individual Differences**

**Part 1 – Model selection**

**Table S1
*Model comparison based on Akaike Information Criterion (AIC) for predictors of PRS_TOT – Praglia sample***

| **Model** | **Predictors** | **df** | **AIC** |
| --- | --- | --- | --- |
| m0 | Intercept only | 2 | 178.94 |
| +m1 | Age | 3 | 180.56 |
| +m2 | SSP_TOT | 3 | 180.11 |
| +m3 | CNS_TOT | 3 | 179.58 |
| +m4 | SCSRF_TOT | 3 | 179.58 |
| +m5 | SIGN_ART & CULTURE | 3 | 159.74 |
| **+m6** | **SIGN_ART & CULTURE, SIGN_RELIGIOSITY** | **4** | **156.51** |
| +m7 | SIGN_ART & CULTURE, SIGN_RELIGIOSITY, SIGN_HISTORY & ANTIQUITY | 5 | 156.71 |
| +m8 | SIGN_ART & CULTURE, SIGN_RELIGIOSITY, SIGN_NATURE | 5 | 155.13 |
|  |  |  |  |

**Table S2
*Model comparison based on Akaike Information Criterion (AIC) for predictors of PRS_fascination – Praglia sample***

| **Model** | **Predictors** | **df** | **AIC** |
| --- | --- | --- | --- |
| m0 | Intercept only | 2 | 178.94 |
| +m1 | Age | 3 | 180.86 |
| +m2 | SSP_TOT | 3 | 179.79 |
| +m3 | CNS_TOT | 3 | 180.87 |
| +m4 | SCSRF_TOT | 3 | 180.66 |
| +m5 | SIGN_ART & CULTURE | 3 | 171.79 |
| +m6 | SIGN_ART & CULTURE, SIGN_RELIGIOSITY | 4 | 173.44 |
| **+m7** | **SIGN_ART & CULTURE, SIGN_HISTORY & ANTIQUITY** | **4** | **167.79** |
| +m8 | SIGN_ART & CULTURE, SIGN_HISTORY & ANTIQUITY, SIGN_NATURE | 5 | 169.27 |

**Table S3
*Model comparison based on Akaike Information Criterion (AIC) for predictors of PRS_being_away – Praglia sample***

| **Model** | **Predictors** | **df** | **AIC** |
| --- | --- | --- | --- |
| m0 | Intercept only | 2 | 178.94 |
| +m1 | Age | 3 | 180.92 |
| +m2 | SSP_TOT | 3 | 180.10 |
| +m3 | CNS_TOT | 3 | 179.36 |
| +m4 | SCSRF_TOT | 3 | 179.28 |
| +m5 | SIGN_ART & CULTURE | 3 | 162.61 |
| **+m6** | **SIGN_ART & CULTURE, SIGN_RELIGIOSITY** | **4** | **159.60** |
| +m7 | SIGN_ART & CULTURE, SIGN_HISTORY & ANTIQUITY | 4 | 160.46 |
| +m8 | SIGN_ART & CULTURE, SIGN_NATURE | 4 | 161.14 |

**Table S4
*Model comparison based on Akaike Information Criterion (AIC) for predictors of PRS_coherence – Praglia sample***

| **Model** | **Predictors** | **df** | **AIC** |
| --- | --- | --- | --- |
| m0 | Intercept only | 2 | 178.94 |
| +m1 | Age | 3 | 180.76 |
| +m2 | SSP_TOT | 3 | 180.08 |
| +m3 | CNS_TOT | 3 | 177.11 |
| +m4 | CNS_TOT, SCSRF_TOT | 4 | 179.91 |
| +m5 | CNS_TOT, SIGN_ART & CULTURE | 4 | 170.43 |
| +m6 | CNS_TOT, SIGN_ART & CULTURE, SIGN_RELIGIOSITY | 5 | 165.57 |
| +m7 | CNS_TOT, SIGN_ART & CULTURE, SIGN_RELIGIOSITY, SIGN_HISTORY & ANTIQUITY | 6 | 167.18 |
| **+m8** | **CNS_TOT, SIGN_ART & CULTURE, SIGN_RELIGIOSITY, SIGN_NATURE** | **6** | **162.03** |

**Table S5
*Model comparison based on Akaike Information Criterion (AIC) for predictors of PRS_scope – Praglia sample***

| **Model** | **Predictors** | **df** | **AIC** |
| --- | --- | --- | --- |
| m0 | Intercept only | 2 | 178.94 |
| +m1 | Age | 3 | 177.19 |
| +m2 | Age, SSP_TOT | 4 | 180.26 |
| +m3 | Age, CNS_TOT | 4 | 180.36 |
| +m4 | Age, SCSRF_TOT | 4 | 179.63 |
| **+m5** | **Age, SIGN_ART & CULTURE** | **4** | **167.11** |
| +m6 | Age, SIGN_ART & CULTURE, SIGN_RELIGIOSITY | 5 | 167.36 |
| +m7 | Age, SIGN_ART & CULTURE, SIGN_HISTORY & ANTIQUITY | 5 | 169.09 |
| +m8 | Age, SIGN_ART & CULTURE, SIGN_NATURE | 5 | 166.22 |

**Table S6
*Model comparison based on Akaike Information Criterion (AIC) for predictors of PRS_TOT – Aquileia sample***

| **Model** | **Predictors** | **df** | **AIC** |
| --- | --- | --- | --- |
| m0 | Intercept only | 2 | 215.83 |
| +m1 | Age | 3 | 217.81 |
| +m2 | SSP_TOT | 3 | 216.06 |
| +m3 | CNS_TOT | 3 | 212.50 |
| +m4 | CNS_TOT, SCSRF_TOT | 4 | 211.26 |
| +m5 | CNS_TOT, SIGN_ART & CULTURE | 4 | 200.47 |
| +m6 | CNS_TOT, SIGN_ART & CULTURE, SIGN_RELIGIOSITY | 5 | 198.85 |
| +m7 | CNS_TOT, SIGN_ART & CULTURE, SIGN_RELIGIOSITY, SIGN_HISTORY & ANTIQUITY | 6 | 200.37 |
| **+m8** | **CNS_TOT, SIGN_ART & CULTURE, SIGN_RELIGIOSITY, SIGN_NATURE** | **6** | **197.32** |

**Table S7
*Model comparison based on Akaike Information Criterion (AIC) for predictors of PRS_fascination – Aquileia sample***

| **Model** | **Predictors** | **df** | **AIC** |
| --- | --- | --- | --- |
| m0 | Intercept only | 2 | 215.83 |
| +m1 | Age | 3 | 217.82 |
| +m2 | SSP_TOT | 3 | 217.83 |
| +m3 | CNS_TOT | 3 | 217.62 |
| +m4 | SCSRF_TOT | 3 | 215.42 |
| **+m5** | **SIGN_ART & CULTURE** | **3** | **206.71** |
| +m6 | SIGN_ART & CULTURE, SIGN_RELIGIOSITY | 4 | 208.24 |
| +m7 | SIGN_ART & CULTURE, SIGN_HISTORY & ANTIQUITY | 4 | 207.47 |
| +m8 | SIGN_ART & CULTURE, SIGN_NATURE | 4 | 207.40 |

**Table S8
*Model comparison based on Akaike Information Criterion (AIC) for predictors of PRS_being_away – Aquileia sample***

| **Model** | **Predictors** | **df** | **AIC** |
| --- | --- | --- | --- |
| m0 | Intercept only | 2 | 215.83 |
| +m1 | Age | 3 | 217.79 |
| +m2 | SSP_TOT | 3 | 213.94 |
| +m3 | SSP_TOT, CNS_TOT | 4 | 209.37 |
| +m4 | SSP_TOT, CNS_TOT, SCSRF_TOT | 5 | 206.81 |
| **+m5** | **SSP_TOT, CNS_TOT, SIGN_ART & CULTURE** | **5** | **202.47** |
| +m6 | SSP_TOT, CNS_TOT, SIGN_ART & CULTURE, SIGN_RELIGIOSITY | 6 | 203.77 |
| +m7 | SSP_TOT, CNS_TOT, SIGN_ART & CULTURE, SIGN_HISTORY & ANTIQUITY | 6 | 202.69 |
| +m8 | SSP_TOT, CNS_TOT, SIGN_ART & CULTURE, SIGN_NATURE | 6 | 203.87 |

**Table S9
*Model comparison based on Akaike Information Criterion (AIC) for predictors of PRS_coherence – Aquileia sample***

| **Model** | **Predictors** | **df** | **AIC** |
| --- | --- | --- | --- |
| m0 | Intercept only | 2 | 215.83 |
| +m1 | Age | 3 | 217.75 |
| +m2 | SSP_TOT | 3 | 217.80 |
| +m3 | CNS_TOT | 3 | 217.36 |
| +m4 | SCSRF_TOT | 3 | 216.75 |
| +m5 | SIGN_ART & CULTURE | 3 | 205.54 |
| +m6 | SIGN_ART & CULTURE, SIGN_RELIGIOSITY | 4 | 203.01 |
| +m7 | SIGN_ART & CULTURE, SIGN_RELIGIOSITY, SIGN_HISTORY & ANTIQUITY | 5 | 204.48 |
| **+m8** | **SIGN_ART & CULTURE, SIGN_RELIGIOSITY, SIGN_NATURE** | **5** | **199.69** |

**Table S10
*Model comparison based on Akaike Information Criterion (AIC) of PRS_scope – Aquileia sample***

| **Model** | **Predictors** | **df** | **AIC** |
| --- | --- | --- | --- |
| m0 | Intercept only | 2 | 215.83 |
| +m1 | Age | 3 | 217.33 |
| +m2 | SSP_TOT | 3 | 216.16 |
| **+m3** | **CNS_TOT** | **3** | **210.78** |
| +m4 | CNS_TOT, SCSRF_TOT | 4 | 212.13 |
| +m5 | CNS_TOT, SIGN_ART & CULTURE | 4 | 211.26 |
| +m6 | CNS_TOT, SIGN_RELIGIOSITY | 4 | 209.60 |
| +m7 | CNS_TOT, SIGN_HISTORY & ANTIQUITY | 4 | 212.74 |
| +m8 | CNS_TOT, SIGN_NATURE | 4 | 210.10 |

**Part 2 – Mixed models**

**Table S11**

***Model comparison based on Akaike Information Criterion (AIC) of SAM_VALENCE – Praglia sample***

| **Model** | **Fixed effects added** | | | | **df** | **AIC** |
| --- | --- | --- | --- | --- | --- | --- |
| m0 | Intercept only | | | | 3 | 326.08 |
| +m1 | + età | | | | 4 | 329.97 |
| +m2 | + SSP_TOT | | | | 4 | 330.38 |
| +m3 | + CNS_TOT | | | | 4 | 327.32 |
| +m4 | + SCSRF_TOT | | | | 4 | 329.13 |
| **+m5** | **+ SIGN_ART & CULTURE** | | | | **4** | **323.68** |
| +m6 | + SIGN_RELIGIOSITY | | | | 5 | 325.75 |
| +m7 | + SIGN_HISTORY & ANTIQUITY | | | | 5 | 325.67 |
| +m8 | + SIGN_NATURE | | | | 5 | 323.69 |
| +m9 | | + time | 5 | 322.69 | | |

**Table S12**

***Model comparison based on Akaike Information Criterion (AIC) of SAM_AROUSAL – Praglia sample***

| **Model** | **Fixed effects added** | **df** | **AIC** |
| --- | --- | --- | --- |
| m0 | Intercept only | 3 | 328.20 |
| +m1 | + età | 4 | 331.28 |
| +m2 | + SSP_TOT | 4 | 332.35 |
| +m3 | + CNS_TOT | 4 | 331.73 |
| +m4 | + SCSRF_TOT | 4 | 330.41 |
| +m5 | + SIGN_ART & CULTURE | 4 | 327.00 |
| **+m6** | **+ SIGN_RELIGIOSITY** | **5** | **324.50** |
| +m7 | + SIGN_HISTORY & ANTIQUITY | 6 | 327.71 |
| +m8 | + SIGN_NATURE | 6 | 328.35 |
| +m9 | + time | 6 | 328.15 |

**Table S13**

***Model comparison based on Akaike Information Criterion (AIC) of SAM_VALENCE – Aquileia sample***

| **Model** | **Fixed effects added** | **df** | **AIC** |
| --- | --- | --- | --- |
| m0 | Intercept only | 3 | 404.13 |
| +m1 | + età | 4 | 408.21 |
| +m2 | + SSP_TOT | 4 | 403.01 |
| +m3 | + CNS_TOT | 4 | 408.44 |
| +m4 | + SCSRF_TOT | 4 | 406.21 |
| +m5 | + SIGN_ART & CULTURE | 4 | 403.05 |
| +m6 | + SIGN_RELIGIOSITY | 5 | 406.25 |
| +m7 | + SIGN_HISTORY & ANTIQUITY | 5 | 407.84 |
| +m8 | + SIGN_NATURE | 5 | 402.67 |
| **+m9** | **+ time** | **5** | **400.96** |

**Table S14**

***Model comparison based on Akaike Information Criterion (AIC) of SAM_AROUSAL – Aquileia sample***

| **Model** | **Fixed effects added** | **df** | **AIC** |
| --- | --- | --- | --- |
| m0 | Intercept only | 3 | 391.77 |
| +m1 | + età | 4 | 395.85 |
| +m2 | + SSP_TOT | 4 | 395.90 |
| +m3 | + CNS_TOT | 4 | 394.86 |
| +m4 | + SCSRF_TOT | 4 | 396.10 |
| +m5 | + SIGN_ART & CULTURE | 4 | 395.65 |
| +m6 | + SIGN_RELIGIOSITY | 4 | 391.64 |
| +m7 | + SIGN_HISTORY & ANTIQUITY | 4 | 395.78 |
| +m8 | + SIGN_NATURE | 4 | 390.09 |
| **+m9** | **+ time** | **5** | **385.27** |

**Table S15. Correlation of Praglia sample**

|  | *(1)* | *(2)* | *(3)* | *(4)* | *(5)* | *(6)* | *(7)* | *(8)* | *(9)* | *(10)* | *(11)* | *(12)* | *(13)* | *(14)* |
| --- | --- | --- | --- | --- | --- | --- | --- | --- | --- | --- | --- | --- | --- | --- |
| *1.Age* |  |  |  |  |  |  |  |  |  |  |  |  |  |  |
| *2.Religiosity (SCSRF)* | 0.097 |  |  |  |  |  |  |  |  |  |  |  |  |  |
| *3.Connectedness (CNS)* | -0.153 | 0.187 |  |  |  |  |  |  |  |  |  |  |  |  |
| *4.Perceived_stress (SSP)* | -0.199 | -0.309^*^ | 0.083 |  |  |  |  |  |  |  |  |  |  |  |
| *5.Restorativeness (PRS-11)* | -0.078 | 0.115 | 0.147 | 0.148 |  |  |  |  |  |  |  |  |  |  |
| *6.Fascination* | -0.036 | -0.068 | -0.034 | 0.136 | 0.777^***^ |  |  |  |  |  |  |  |  |  |
| *7.Being_away* | 0.016 | 0.163 | 0.159 | 0.116 | 0.870^***^ | 0.507^***^ |  |  |  |  |  |  |  |  |
| *8.Coherence* | -0.053 | 0.128 | 0.245 | 0.117 | 0.838^***^ | 0.526^***^ | 0.688^***^ |  |  |  |  |  |  |  |
| *9.Scope* | -0.242 | 0.144 | 0.097 | 0.105 | 0.702^***^ | 0.463^***^ | 0.476^***^ | 0.451^***^ |  |  |  |  |  |  |
| *10.Significance tot* | -0.062 | 0.213 | 0.039 | -0.030 | 0.569^***^ | 0.349^**^ | 0.474^***^ | 0.556^***^ | 0.461^***^ |  |  |  |  |  |
| *11.Sign_Art&culture* | -0.170 | 0.022 | 0.037 | 0.003 | 0.538^***^ | 0.370^**^ | 0.506^***^ | 0.395^**^ | 0.447^***^ | 0.598^***^ |  |  |  |  |
| *12.Sign_Religiosity* | 0.148 | 0.498^***^ | 0.101 | -0.307^*^ | 0.347^**^ | 0.146 | 0.341^**^ | 0.374^**^ | 0.240 | 0.604^***^ | 0.210 |  |  |  |
| *13.Sign_Hystory&antiquity* | -0.034 | 0.143 | -0.044 | -0.075 | 0.523^***^ | 0.463^***^ | 0.483^***^ | 0.383^**^ | 0.314^*^ | 0.760^***^ | 0.728^***^ | 0.316^*^ |  |  |
| *14.Sign_Nature* | -0.009 | 0.084 | 0.156 | -0.023 | 0.447^***^ | 0.299^*^ | 0.335^**^ | 0.468^***^ | 0.350^**^ | 0.707^***^ | 0.390^**^ | 0.392^**^ | 0.488^***^ |  |
| *Computed correlation used pearson-method with listwise-deletion.* | | | | | | | | | | | | | | |

**Table S16. Correlation of Aquileia sample**

|  | *(1)* | *(2)* | *(3)* | *(4)* | *(5)* | *(6)* | *(7)* | *(8)* | *(9)* | *(10)* | *(11)* | *(12)* | *(13)* | *(14)* |
| --- | --- | --- | --- | --- | --- | --- | --- | --- | --- | --- | --- | --- | --- | --- |
| *1.Age* |  |  |  |  |  |  |  |  |  |  |  |  |  |  |
| *2.Religiosity (SCSRF)* | 0.236^*^ |  |  |  |  |  |  |  |  |  |  |  |  |  |
| *3.Connectedness (CNS)* | 0.017 | 0.176 |  |  |  |  |  |  |  |  |  |  |  |  |
| *4.Perceived_stress (SSP)* | -0.161 | -0.082 | 0.091 |  |  |  |  |  |  |  |  |  |  |  |
| *5.Restorativeness (PRS-11)* | 0.017 | 0.241^*^ | 0.262^*^ | 0.153 |  |  |  |  |  |  |  |  |  |  |
| *6.Fascination* | -0.015 | 0.178 | 0.053 | -0.003 | 0.646^***^ |  |  |  |  |  |  |  |  |  |
| *7.Being_away* | 0.024 | 0.255^*^ | 0.302^**^ | 0.225 | 0.857^***^ | 0.392^***^ |  |  |  |  |  |  |  |  |
| *8.Coherence* | -0.033 | 0.120 | 0.079 | 0.022 | 0.778^***^ | 0.428^***^ | 0.507^***^ |  |  |  |  |  |  |  |
| *9.Scope* | 0.082 | 0.140 | 0.300^**^ | 0.149 | 0.635^***^ | 0.309^**^ | 0.417^***^ | 0.303^**^ |  |  |  |  |  |  |
| *10.Significance tot* | 0.163 | 0.315^**^ | 0.206 | 0.074 | 0.468^***^ | 0.309^**^ | 0.367^**^ | 0.423^***^ | 0.273^*^ |  |  |  |  |  |
| *11.Sign_Art&culture* | -0.048 | 0.129 | -0.022 | 0.018 | 0.393^***^ | 0.371^**^ | 0.284^*^ | 0.389^***^ | 0.129 | 0.491^***^ |  |  |  |  |
| *12.Sign_Religiosity* | 0.270^*^ | 0.315^**^ | 0.159 | -0.042 | 0.314^**^ | 0.152 | 0.227 | 0.302^**^ | 0.240^*^ | 0.809^***^ | 0.217 |  |  |  |
| *13.Sign_Hystory&antiquity* | 0.003 | 0.001 | -0.201 | -0.003 | 0.061 | 0.175 | 0.117 | -0.034 | -0.083 | 0.307^**^ | 0.153 | -0.056 |  |  |
| *14.Sign_Nature* | 0.024 | 0.220 | 0.395^***^ | 0.225 | 0.401^***^ | 0.196 | 0.311^**^ | 0.373^***^ | 0.283^*^ | 0.736^***^ | 0.206 | 0.394^***^ | 0.084 |  |
| *Computed correlation used pearson-method with listwise-deletion.* | | | | | | | | | | | | | | |
